# Bio-tolerance potential and environmental risks assessment of *Oreochromis niloticus* and *Ipomoea aquatica* in Agodi Reservoir, Nigeria

**DOI:** 10.1038/s41598-022-05576-2

**Published:** 2022-01-31

**Authors:** P. O. Ogungbile, A. O. Ajibare, P. O. Ayeku, J. O. Akinola

**Affiliations:** 1 Environmental Management and Crop Production Unit, College of Agriculture Engineering and Science, Bowen University Iwo, Iwo, Nigeria; 2 Department of Fisheries and Aquaculture Technology, Olusegun Agagu University of Science and Technology Okitipupa, Okitipupa, Nigeria; 3 Department of Biological Sciences, Federal University Gusau, Gusau, Nigeria; 4 Department of Fisheries and Aquaculture Technology, Federal University of Technology Akure, Akure, Nigeria

**Keywords:** Biological techniques, Environmental sciences, Natural hazards

## Abstract

The concentration of nine heavy metals in *Oreochromis niloticus* and *Ipomoea aquatica* inhabiting Agodi reservoir, Oyo State, Nigeria were investigated for twelve months. The concentrations of the metals were carried out using PG990 Atomic Absorption Spectrophotometer. The Ecological Risk Quotient (ERQ) was calculated using standard methods while the tolerability was determined with Box Plot analysis. The metal bioaccumulation in *O. niloticus* and *I. aquatica* followed the order Mn > Fe > Zn > Cu > Co > Pb > Cd > Cr > Ni and Fe > Zn > Mn > Cu > Pb > Cd > Co > Cr > Ni respectively. The results revealed *O. niloticus* to bioaccumulate the metals more than *I. aquatica.* Most of the examined metals were higher than the safety limit for the metals concentration in *O. niloticus* and *I. aquatica*. Also, *I. aquatica* had higher tolerability for heavy metals than *O. niloticus.* In *O. niloticus*, metal concentration, as well as ERQ, was higher in the wet season while no particular order was observed for *I. aquatica.* The ERQ result revealed that Cd, Fe, Mn, Zn, Cu and Pb exhibited a high level of ecological risk to both the aquatic flora and fauna as the ERQ values were above the risk limit of one (1). Thus, there is a significant environmental risk associated with heavy metals in the water body.

## Introduction

Heavy metals are pollutants that are non-biodegradable and are persistent in the environment. They enter the aquatic environments through various anthropogenic activities, industrialization, urbanization and extensive use of chemicals in agriculture^1^, inevitably disrupting and contaminating the aquatic food chain. They are toxic to living organisms with great potential to bio-accumulate in various organs of the body, causing severe harm to the human body system^2^. These metals threaten human health, plant growth and the survival of animal life through the disruption and destruction of vital biochemical processes in the ecosystem^3^.

*Oreochromis niloticus* (Nile Tilapia) is a widely distributed, abundant and economically important sedentary fish present in most freshwater ecosystem. It is often used as a bio-indicator of measuring the pollution level of an aquatic environment^4^. *Ipomoea aquatica* also known as water spinach is a herbaceous trailing vine that grows perennially and is widely distributed. It is economically important and mostly cultivated by both rural and urban dwellers due to its nutritional composition and benefits. It is a vital component of daily meals with the stems and leaves used as vegetables in the human diet. It supplies high minerals such as iron, Vitamins A, C and E, fibres which are required for healthy living and also serves medicinal purposes^5^. *Ipomoea aquatica*, one of the cheapest vegetables and staple foods in Nigeria, is mostly grown and harvested from polluted wetland and aquatic ecosystems^6^.

Researches from various authors have shown that aquatic plants possess the ability to both retain and remove metals from contaminated water bodies^6–8^. The ability of *I. aquatica* to bio-concentrate and bio-accumulate metals at concentration 100,000 times greater than water and the potential of more contamination when compared with other plants^9,10^ makes this study imperative.

Although vegetables and fish are an important component of the daily diet of humans with high nutritional value^11^, there is a paucity of information on its contamination by heavy metals in Agodi Reservoir as their consumption in contaminated form can lead to adverse health problems in animals and humans with deleterious consequences. Hence, the need to examine and accentuate the bioconcentrations and tolerability of heavy metals in the aquatic fauna and flora inhabiting Agodi Reservoir in Nigeria.

## Materials and methods

### Study area

Agodi Reservoir (longitude 3° 56′ 18″ E and latitude 7° 26′ 11″ N) was formed out of River Ogunpa in Ibadan, Oyo State Nigeria. It has an area of about 5.2 hectares, stretching about 1 km with a depth of about 5 m. The study area has two major seasons; the dry season and the wet season and experiences consistently high temperatures throughout the year^6^.

### Collection and identification of samples

*Oreochromis niloticus* (with average weight and length of 204.65 ± 14.86 g and 15.50 ± 3.20 cm respectively) and *Ipomoea aquatica* were collected monthly from the Agodi Reservoir in Ibadan, Oyo State Nigeria for twelve months. Samples were collected from all the locations on the same day with the assistance of artisanal fishermen. The plant samples were collected with 2 m offset from the watercourse for all the locations. The whole plant containing the leaves, flowers, stem and roots were thoroughly cleaned with ‘fresh’ water followed by distilled water for quantitative removal of soil and other foreign particles and kept separately in polyethylene bags before being transported to the herbarium of the Botany Department, of the University of Ibadan, Nigeria. *O. niloticus* was identified according to^12,13^. Fish samples from each sampling point were kept separately in labelled polyethylene bags and transported in an ice chest to the laboratory.

### Determination of heavy metals

Samples (i.e. fish muscle and whole plant) were oven-dried at 60 °C for 48 h and were digested according to^14^ in the research laboratory of the Department of Chemistry, Bowen University Iwo, Nigeria. The concentrations of heavy metals were then determined with PG990 Atomic Absorption Spectrophotometer. The heavy metals that were assessed include Cd, Co, Cr, Cu Fe, Mn, Ni, Pb and Zn.

Double distilled deionized water was used throughout the analyses, i.e. glassware and plastic ware (Merck, Germany) used were thoroughly rinsed with 10% HNO_3_ followed by washing with de-ionized distilled water. For quality control data assurance each sample was analyzed in triplicate. Analytical blanks were run in the same way as the samples and concentrations were determined using standard solutions prepared in the same acid matrix. Standards for the instrument calibration were prepared on the basis of monoelement certified reference solution ICP Standard (Merck, Germany).

The experimental methods and procedures were performed in accordance with the guidelines and regulations of the Fisheries and Aquaculture Laboratory, Federal University of Technology, Akure Nigeria and approved by the University’s Committee on Animal Science. The study was also carried out in compliance with the Animal Research: Reporting of In Vivo Experiments (ARRIVE) guidelines.

### Statistical analysis

Box Plot was used to determine the tolerability of heavy metals in the fish and plant (at P = 0.05) using SPSS 20.0. The tolerability (which is the extent to which organisms can withstand the accumulation of heavy metals) was presented graphically with box plot, in which each box indicated the 25th and 75th percentile values while bold line depicts the mean value, the error bars denote the standard deviation, the open circle and asterisk values indicated the threshold and concentration at which the organisms could not tolerate the metals. Ecological Risk Quotient (the numerically evaluated associated risks for quantification and interpretation of chemical concentrations in the aquatic media) was calculated as^15^:

$${\mathrm{ERQ}}=\frac{Environmental\, Concentration\, (mg/kg)}{Recommended\, Limit\, (mg/kg)}$$

### Animal welfare statement

The authors confirm that the ethical policies of the journal, as noted on the journal’s author guidelines page, have been adhered to and the appropriate ethical review committee approval of both the Fisheries and Aquaculture Department and the Central Research Laboratory, Federal University of Technology Akure, Nigeria has been received. The authors confirm that they have followed EU standards for the protection of animals used for scientific purposes.

## Results

### The concentration of heavy metals in *O. niloticus* in Agodi Reservoir

The concentration of heavy metals in *O. niloticus* is presented in Table 1. From the table, the metal bioaccumulation followed the order Mn > Fe > Zn > Cu > Co > Pb > Cd > Cr > Ni. All the metals excluding Co, Ni and Cr were higher than the threshold limits of heavy metal contamination in fishes^25^(Table 1). Also, the bioaccumulation of metals was higher during the wet season than the dry season, except for Cd, Co and Ni. The mean concentration of Cr, Cu, Fe, Mn, Pb and Zn from dry season to wet season ranged from 0.00 ± 0.00 to 2.18 ± 9.54, 5.54 ± 4.78 to 13.64 ± 18.64, 266.63 ± 692.31 to 429.10 ± 532.69, 250.75 ± 484.94 to 477.78 ± 762.58, 0.32 ± 1.25 to 2.18 ± 8.34 and 186.38 ± 219.21 to 206.63 ± 183.45 mg/kg respectively.

**Table 1:** Concentration of Heavy Metals (mg/kg) in *O. niloticus* in Agodi Reservoir.

Month/Season	Cd	Co	Cr	Cu	Fe	Mn	Ni	Pb	Zn
January	0.00 ± 0.00	0.00 ± 0.00	0.00 ± 0.00	1.36 ± 0.45	0.00 ± 0.00	63.33 ± 15.42	0.00 ± 0.00	0.00 ± 0.00	112.19 ± 41.12
February	0.00 ± 0.00	0.00 ± 0.00	0.00 ± 0.00	5.19 ± 3.93	0.00 ± 0.00	16.00 ± 27.71	0.00 ± 0.00	0.00 ± 0.00	37.85 ± 36.50
March	0.23 ± 0.40	0.00 ± 0.00	0.00 ± 0.00	4.22 ± 3.20	422.50 ± 730.49	21.83 ± 18.13	0.00 ± 0.00	1.79 ± 3.10	121.89 ± 44.54
April	0.00 ± 0.00	0.00 ± 0.00	0.00 ± 0.00	3.73 ± 0.92	602.75 ± 0.00	43.17 ± 8.86	3.46 ± 3.24	0.00 ± 0.00	61.13 ± 2.93
May	0.47 ± 0.48	1.30 ± 2.25	0.64 ± 1.11	27.42 ± 33.94	637.00 ± 647.23	1251.92 ± 1100.14	0.00 ± 0.00	13.50 ± 21.38	382.34 ± 124.41
June	0.00 ± 0.00	2.06 ± 1.10	14.58 ± 25.26	33.93 ± 23.40	409.17 ± 517.91	986.50 ± 1049.65	0.00 ± 0.00	0.00 ± 0.00	91.53 ± 74.44
July	0.00 ± 0.00	2.27 ± 1.22	0.00 ± 0.00	20.23 ± 11.81	896.83 ± 673.03	921.93 ± 890.97	0.00 ± 0.00	0.00 ± 0.00	365.82 ± 324.23
August	0.00 ± 0.00	2.70 ± 2.42	0.00 ± 0.00	8.93 ± 3.09	1332.33 ± 1107.07	1003.08 ± 756.67	3.39 ± 5.87	1.61 ± 0.00	446.29 ± 398.49
September	0.00 ± 0.00	0.00 ± 0.00	0.00 ± 0.00	3.75 ± 5.76	35.25 ± 39.43	41.00 ± 26.38	1.02 ± 1.11	0.00 ± 0.00	114.71 ± 178.59
October	0.00 ± 0.00	0.00 ± 0.00	0.00 ± 0.00	2.19 ± 0.59	0.17 ± 0.14	78.08 ± 65.85	0.00 ± 0.00	0.00 ± 0.00	309.02 ± 37.40
November	0.18 ± 0.32	14.75 ± 25.55	0.00 ± 0.00	3.59 ± 3.28	0.83 ± 1.44	124.92 ± 22.12	1.93 ± 3.33	0.00 ± 0.00	100.28 ± 128.88
December	11.69 ± 20.25	0.00 ± 0.00	0.00 ± 0.00	8.61 ± 7.80	0.00 ± 0.00	46.42 ± 36.78	0.00 ± 0.00	0.00 ± 0.00	235.26 ± 23.77
Dry Season	2.38 ± 9.05	3.49 ± 11.37	0.00 ± 0.00	5.54 ± 4.78	266.63 ± 692.31	250.75 ± 484.94	1.06 ± 2.93	0.32 ± 1.25	186.38 ± 219.21
Wet Season	0.10 ± 0.26	0.80 ± 1.33	2.18 ± 9.54	13.64 ± 18.64	429.10 ± 532.69	477.78 ± 762.58	0.64 ± 1.64	2.18 ± 8.34	206.63 ± 183.45
25	0.50	2.00	0.50	3.00	30.00	0.50	5.00	2.30	67.90
16	0.20	2.00	0.50	3.00	30.00	0.50	–	–	–

Source: Field Survey. (The values in the bottom rows are threshold values obtained from regulatory standards according to FAO/WHO^16^ and WHO^25^).

### Ecological risks of heavy metals in *O. niloticus* in Agodi Reservoir

The ERQ of heavy metals in *O. niloticus* is presented in Table 2. The Table showed that all the metals except for Co, Cr and Ni exhibited a high level of ecological risk to the aquatic biota as the ERQ values were above one (1). The ERQ of Fe (533.27 and 858.19) and Mn (501.50 and 955.55) in the dry and wet seasons respectively were extremely high when compared with the other metals that had high ERQ (Table 2). Zn, Cu and Pb also had ERQ values of 6.89, 4.55 and 1.09 respectively in the wet season, while Zn, Cd and Cu had ERQ values of 6.21, 4.75 and 1.85 during the dry season. Also, high ERQ values were recorded for Zn and Mn across the study period. Also, ERQ values were generally high in the wet season than the dry season except Cd, Co and Ni (Table 2).

**Table 2:** Ecological Risk Quotient of Heavy Metals in *O. niloticus* in Agodi Reservoir.

Month/Season	Cd	Co	Cr	Cu	Fe	Mn	Ni	Pb	Zn
January	0.00	0.00	0.00	0.45	0.00	126.67*	0.00	0.00	3.74*
February	0.00	0.00	0.00	1.73*	0.00	32.00*	0.00	0.00	1.26*
March	0.47	0.00	0.00	1.41*	845.00*	43.67*	0.00	0.90	4.06*
April	0.00	0.00	0.00	1.24*	1205.50*	86.33*	0.05	0.00	2.04*
May	0.93	0.26	0.28	9.14*	1274.00*	2503.83*	0.00	6.75*	12.74*
June	0.00	0.41	6.34*	11.31*	818.33*	1973.00*	0.00	0.00	3.05*
July	0.00	0.45	0.00	6.74*	1793.67*	1843.87*	0.00	0.00	12.19*
August	0.00	0.54	0.00	2.98*	2664.67*	2006.17*	0.05	0.80	14.88*
September	0.00	0.00	0.00	1.25*	70.50*	82.00*	0.01	0.00	3.82*
October	0.00	0.00	0.00	0.73	0.33	156.17*	0.00	0.00	10.30*
November	0.37	2.95*	0.00	1.20*	1.67*	249.83*	0.03	0.00	3.34*
December	23.38*	0.00	0.00	2.87*	0.00	92.83*	0.00	0.00	7.84*
Dry Season	4.75*	0.70	0.00	1.85*	533.27*	501.50*	0.02	0.16	6.21*
Wet Season	0.20	0.16	0.95	4.55*	858.19*	955.55*	0.01	1.09*	6.89*

ERQ values with asterisk ‘*’ are above the ecological risk limit. (Source: Field Survey).

### The concentration of heavy metals in *I. aquatic* in Agodi Reservoir

The concentration of heavy metals in *I. aquatica* is presented in Table 3. The table showed that the metal bioaccumulation followed the order Fe > Zn > Mn > Cu > Pb > Cd > Co > Cr > Ni. Some of the metals (Cd, Cu, Fe, Mn and Pb) were higher than the threshold limits^25^ of heavy metal contamination in plants while Co, Cr, Ni and Zn were lower than the threshold limit (Table 3). Also, the bioaccumulation of Cr, Fe, Ni and Pb were higher during the wet season than the dry season, while Co, Cu, Mn and Zn were higher during the dry season than the wet season. The mean concentration of Co, Cr, Cu, Fe, Mn, Ni, Pb and Zn from dry season to wet season range from 0.21 ± 0.20 to 0.13 ± 0.23, 0.00 ± 0.00 to 0.14 ± 0.38, 7.60 ± 2.46 to 5.10 ± 3.55, 46.28 ± 54.39 to 288.40 ± 715.58, 32.97 ± 62.66 to 17.68 ± 41.87, 0.00 ± 0.00 to 0.06 ± 0.14, 0.00 ± 0.00 to 13.30 ± 35.19 and 46.13 ± 74.74 to 17.30 ± 9.59 mg/kg respectively (Table 3).

**Table 3:** Concentration of Heavy Metals (mg/kg) in *I. aquatic* in Agodi Reservoir.

Month/Season	Cd	Co	Cr	Cu	Fe	Mn	Ni	Pb	Zn
January	0.30 ± 0.36	0.18 ± 0.30	0.00 ± 0.00	4.58 ± 4.28	7.70 ± 6.12	7.26 ± 12.42	0.00 ± 0.00	0.00 ± 0.00	6.79 ± 1.97
February	0.00 ± 0.00	0.38 ± 0.66	0.00 ± 0.00	9.39 ± 12.17	135.28 ± 226.88	144.75 ± 242.17	0.00 ± 0.00	0.00 ± 0.00	178.27 ± 131.99
March	0.00 ± 0.00	0.10 ± 0.17	0.00 ± 0.00	3.15 ± 1.87	4.45 ± 5.06	0.51 ± 0.20	0.00 ± 0.00	0.00 ± 0.00	13.53 ± 1.87
April	1.10 ± 1.91	0.00 ± 0.00	1.00 ± 1.73	3.38 ± 1.09	1910.50 ± 442.81	112.25 ± 92.92	0.00 ± 0.00	0.00 ± 0.00	26.81 ± 2.88
May	0.43 ± 0.05	0.01 ± 0.01	0.00 ± 0.00	0.00 ± 0.00	39.92 ± 1.77	0.08 ± 0.14	0.38 ± 0.65	0.00 ± 0.00	7.53 ± 2.18
June	0.07 ± 0.12	0.65 ± 1.13	0.00 ± 0.00	10.37 ± 1.89	0.00 ± 0.00	0.25 ± 0.43	0.00 ± 0.00	0.00 ± 0.00	3.06 ± 2.11
July	0.00 ± 0.00	0.11 ± 0.19	0.00 ± 0.00	7.37 ± 5.24	0.00 ± 0.00	0.09 ± 0.08	0.00 ± 0.00	0.00 ± 0.00	19.99 ± 4.88
August	0.99 ± 0.54	0.03 ± 0.01	0.00 ± 0.00	10.76 ± 0.74	32.50 ± 17.35	1.33 ± 1.53	0.01 ± 0.01	0.00 ± 0.00	8.07 ± 2.84
September	0.07 ± 0.09	0.01 ± 0.01	0.00 ± 0.00	7.86 ± 4.40	11.08 ± 16.46	0.25 ± 0.25	0.04 ± 0.07	0.00 ± 0.00	21.93 ± 17.86
October	0.30 ± 0.26	0.04 ± 0.01	0.00 ± 0.00	3.54 ± 1.00	52.83 ± 1.61	10 .33 ± 2.57	0.00 ± 0.00	93.10 ± 161.25	28.26 ± 3.15
November	0.13 ± 0.20	0.00 ± 0.00	0.00 ± 0.00	6.56 ± 2.32	55.92 ± 4.19	11.50 ± 1.09	0.00 ± 0.00	0.00 ± 0.00	32.71 ± 7.35
December	0.00 ± 0.00	0.43 ± 0.75	0.00 ± 0.00	6.72 ± 6.59	0.00 ± 0.00	0.00 ± 0.00	0.01 ± 0.01	0.00 ± 0.00	4.79 ± 2.06
Dry Season	0.28 ± 0.41	0.21 ± 0.20	0.00 ± 0.00	7.60 ± 2.46	46.28 ± 54.39	32.97 ± 62.66	0.00 ± 0.00	0.00 ± 0.00	46.13 ± 74.74
Wet Season	0.28 ± 0.40	0.13 ± 0.23	0.14 ± 0.38	5.10 ± 3.55	288.40 ± 715.58	17.68 ± 1.87	0.06 ± 0.14	13.30 ± 35.19	17.30 ± 9.59
25	0.20	0.30	20.00	73.50	99.40	20.00	5.00	2.30	67.90

Source: Field Survey (The values in the bottom row are threshold values obtained from regulatory standards according to WHO^25^).

### Ecological risks of heavy metals in plant in Agodi Reservoir

The ERQ of heavy metals in *I. aquatica* is presented in Table 4. From the table, Cd, Fe, Mn and Pb pose a high ecological risk to the aquatic plant as their ERQ values were above the safety limit of one (1), while Co, Cr, Cu, Ni and Zn had no ecological risk and effect on the plant species as their ERQ values were below the risk limit of one (1).

**Table 4:** Ecological Risk Quotient of Heavy Metals in *I. aquatic* in Agodi Reservoir.

Month/Season	Cd	Co	Cr	Cu	Fe	Mn	Ni	Pb	Zn
January	1.50*	0.04	0.00	0.06	0.39	0.36	0.00	0.00	0.07
February	0.00	0.08	0.00	0.13	6.76*	7.24*	0.00	0.00	1.79*
March	0.00	0.02	0.00	0.04	0.22	0.03	0.00	0.00	0.14
April	5.50*	0.00	0.43	0.05	95.53*	5.61*	0.00	0.00	0.27
May	2.13*	0.00	0.00	0.00	2.00*	0.00	0.01	0.00	0.08
June	0.33	0.13	0.00	0.14	0.00	0.01	0.00	0.00	0.03
July	0.00	0.02	0.00	0.10	0.00	0.00	0.00	0.00	0.20
August	4.96*	0.01	0.00	0.15	1.63*	0.07	0.00	0.00	0.08
September	0.33	0.00	0.00	0.11	0.55	0.01	0.00	0.00	0.22
October	1.50*	0.01	0.00	0.05	2.64*	0.52	0.00	310.33*	0.28
November	0.63	0.00	0.00	0.09	2.80*	0.58	0.00	0.00	0.33
December	0.00	0.09	0.00	0.09	0.00	0.00	0.00	0.00	0.05
Dry Season	1.42	0.04	0.00	0.10	2.31	1.65	0.00	0.00	0.46
Wet Season	1.40	0.03	0.06	0.07	14.42	0.88	0.00	44.33	0.17

ERQ values with asterisk ‘*’ are above the ecological risk limit (Source: Field Survey).

The ERQ of Fe (533.27 and 858.19) and Mn (501.50 and 955.55) in the dry and wet seasons respectively were extremely high when compared with the other metals that had high ERQ (Table 4). Zn, Cu and Pb also had ERQ values of 6.89, 4.55 and 1.09 respectively in the wet season, while Zn, Cd and Cu had ERQ values of 6.21, 4.75 and 1.85 during the dry season. Also, high ERQ values were recorded for Zn and Mn across the study period, while Fe and Cu were also almost obtained all through the period of study. In addition, ERQ values were generally high in the dry season than the wet season except Cr, Fe, Mn and Pb (Table 4).

### Tolerability of heavy metals in *Oreochromis niloticus* and *Ipomoea aquatic*

**Figure 1:**
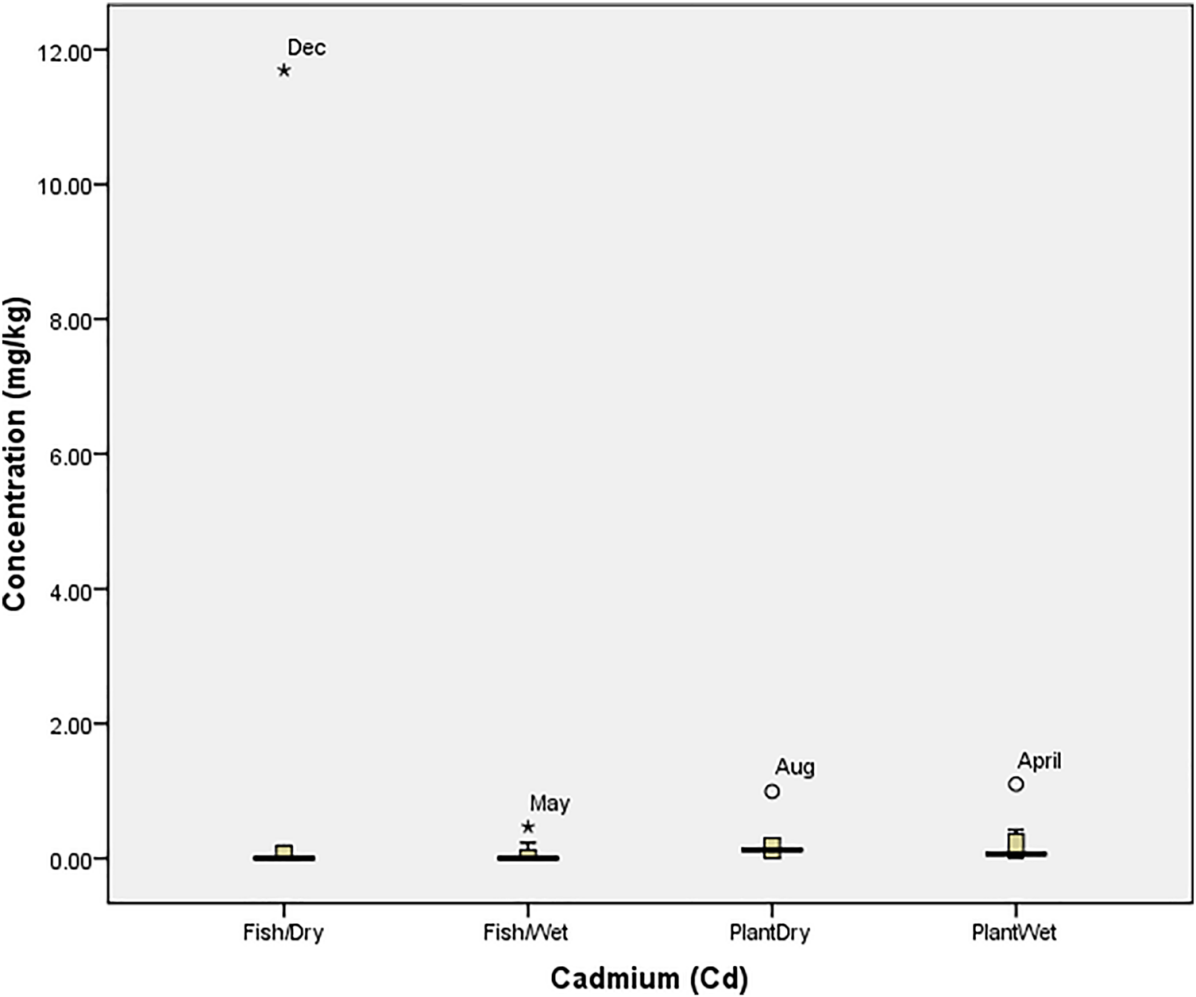
Tolerability of Cadmium in *O. niloticus* (Fish) and *I. aquatica* (Plant) in Wet and Dry Seasons (Source: Field Survey).

**Figure 2:**
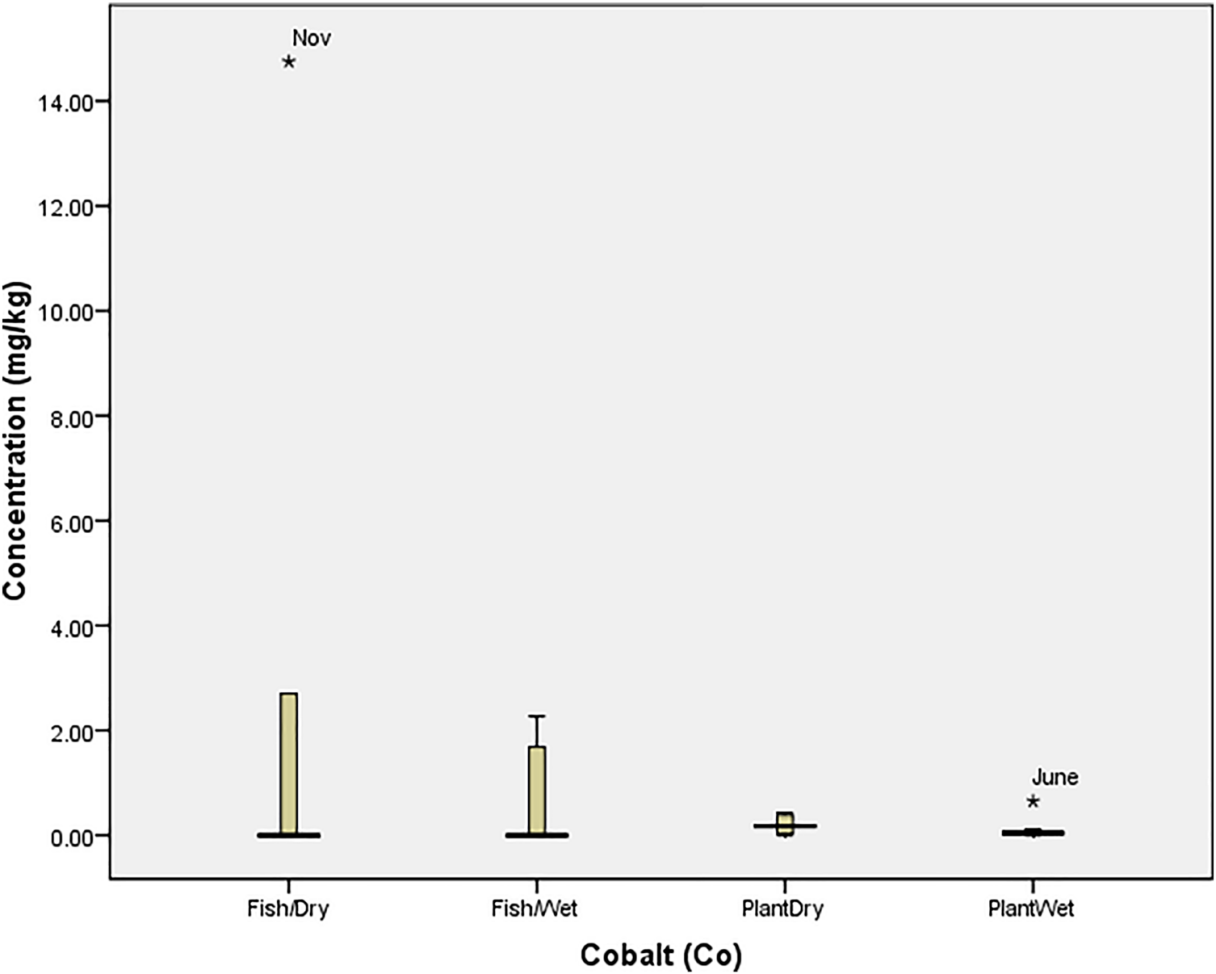
Tolerability of Cobalt in *O. niloticus* (Fish) and *I. aquatica* (Plant) in Wet and Dry Seasons (Source: Field Survey).

**Figure 3:**
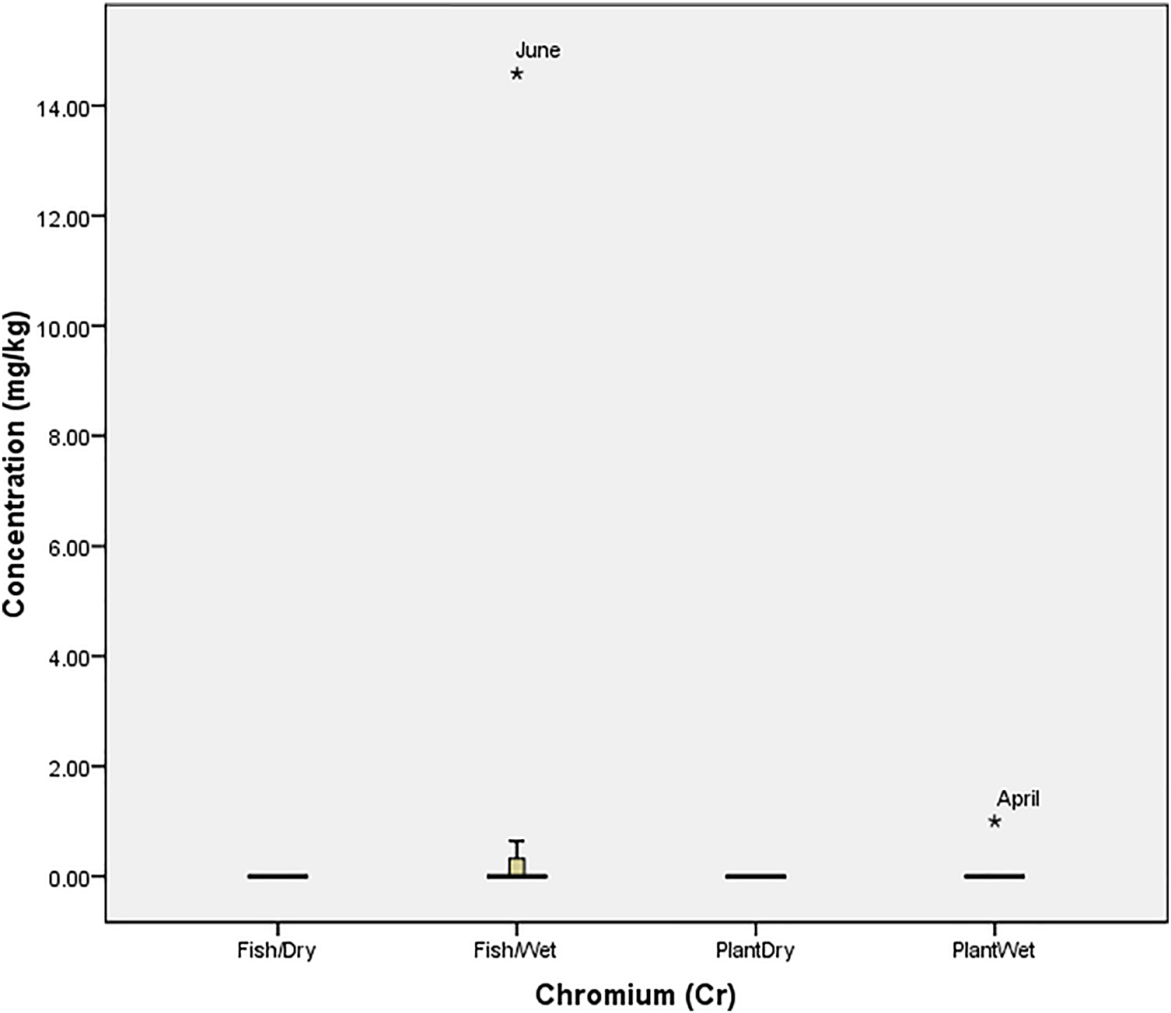
Tolerability of Chromium in *O. niloticus* (Fish) and *I. aquatica* (Plant) in Wet and Dry Seasons (Source: Field Survey).

The tolerability of *O. niloticus* (Fish) and *I. aquatica* (Plant) to cadmium (Cd) in the wet and dry seasons is presented in Fig. 1. The figure revealed that the tolerability of fish spiked in the dry season (11.69 mg/kg in December) and wet season (0.47 mg/kg in May) while *I. aquatica* was at threshold in August (0.99 mg/kg) and April (1.10 mg/kg). Similarly, Fig. 2 showed that the concentrations of cobalt (Co) was only beyond the tolerability of *O. niloticus* in (November; 14.75 mg/kg) the dry season while *I. aquatica* experienced a spike (0.65 mg/kg) in June (Wet season). According to Fig. 3, the concentration of chromium (Cr) was not above the threshold of both *O. niloticus* and *I. aquatica* in the dry season but the concentration was beyond the tolerability of *O. niloticus* (14.58 mg/kg in June) and *I. aquatica* (1.00 mg/kg in April) in the wet season.

**Figure 4:**
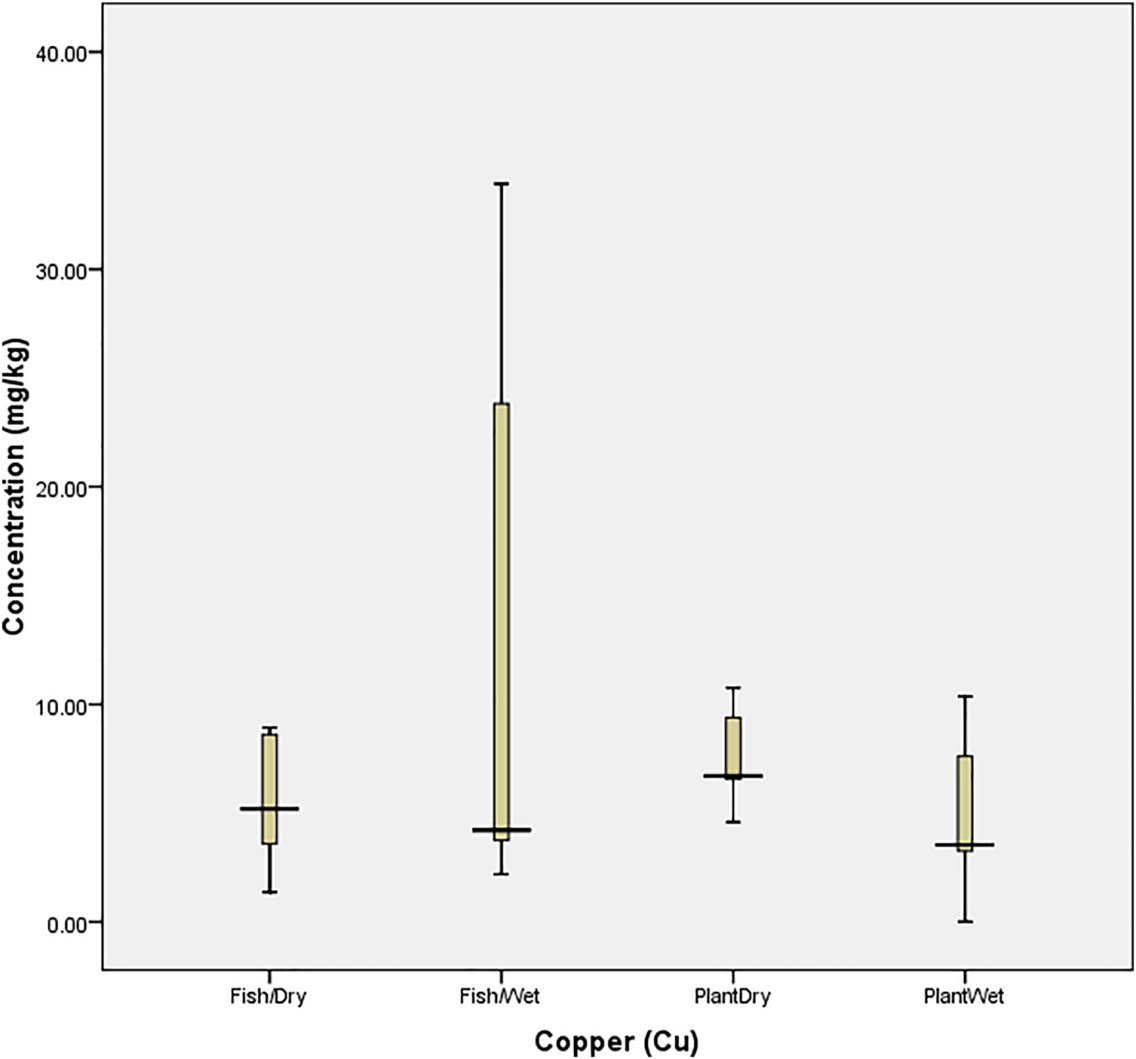
Tolerability of Copper (Cu) in *O. niloticus* (Fish) and *I. aquatica* (Plant) in Wet and Dry Seasons (Source: Field Survey).

**Figure 5:**
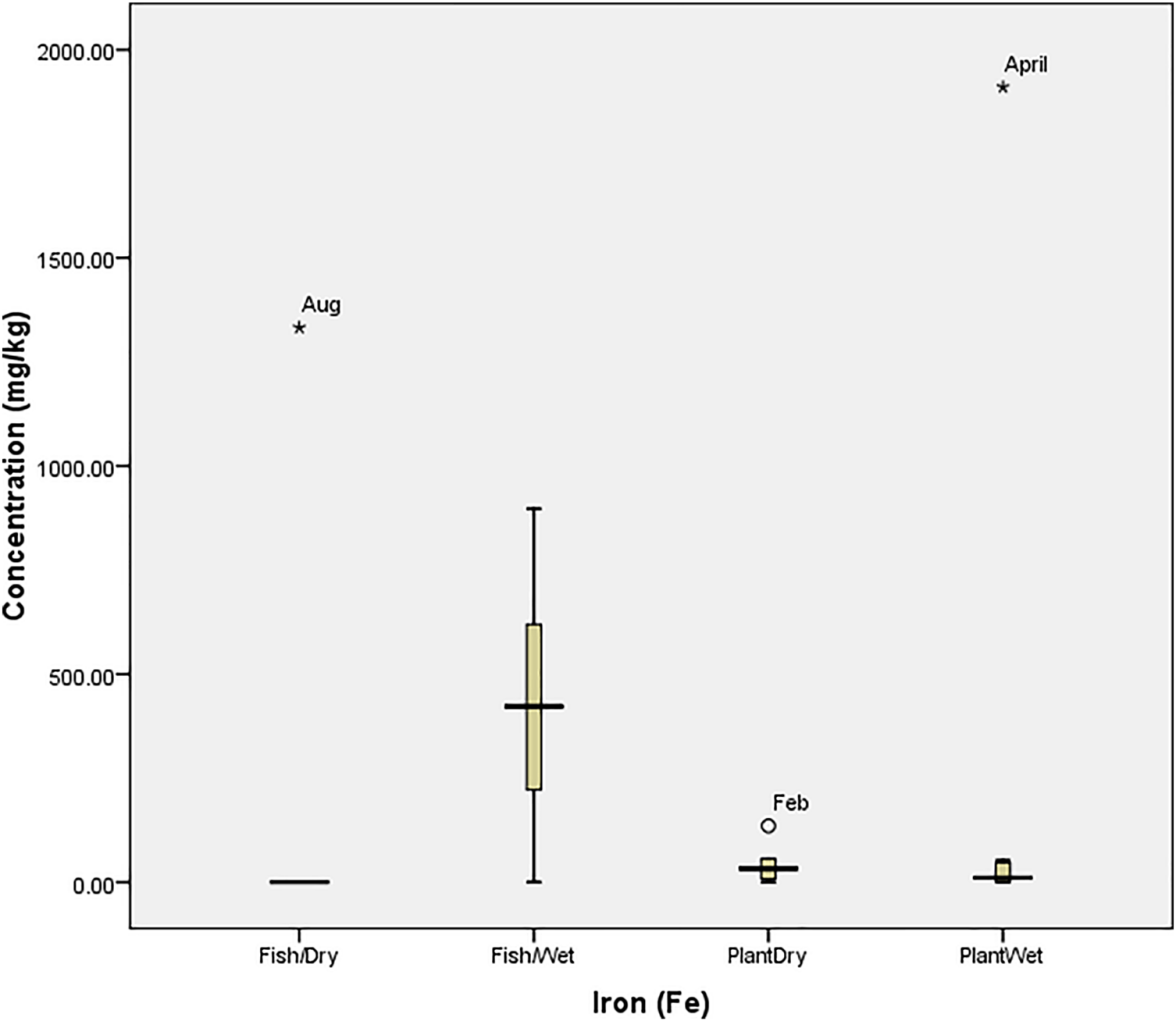
Tolerability of Iron (Fe) in *O. niloticus* (Fish) and *I. aquatica* (Plant) in Wet and Dry Seasons (Source: Field Survey).

**Figure 6:**
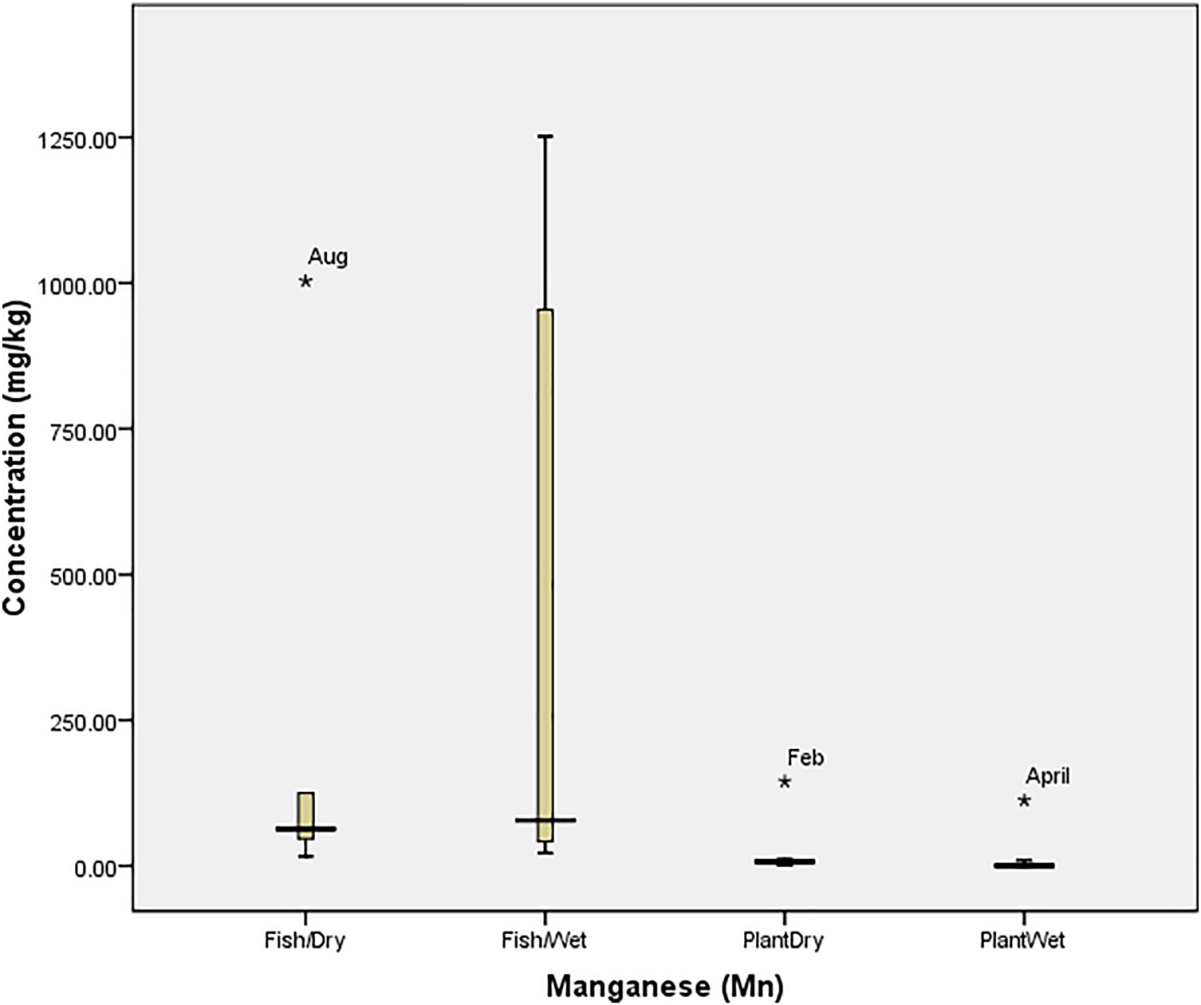
Tolerability of Manganese (Mn) in *O. niloticus* (Fish) and *I. aquatica* (Plant) in Wet and Dry Seasons (Source: Field Survey).

Also, Fig. 4 revealed that the concentration of Cu in both the fish and plant were within the tolerance of the organisms. Figure 5 also revealed that *O. niloticus* did not bio-accumulate Fe beyond the tolerance limit in the wet season. However, it experienced a spike in August (1332.33 mg/kg). Similarly, the concentration in *I. aquatica* was at threshold (135.8 mg/kg in February) but was beyond the tolerability of the plant in April (1910.50 mg/kg); a wet season. The bio-tolerance of *O. niloticus* (Fish) and *I. aquatica* (Plant) to manganese (Mn) in the wet and dry seasons (as presented in Fig. 6) revealed that no concentration was beyond the tolerability of *O. niloticus*, despite the high concentration recorded during the wet season months. However, the concentration (1003.08 mg/kg) recorded in August (Dry season) was beyond the tolerance limit of the fish. The figure also revealed that the values recorded in February (144.75 mg/kg) and April (112.25 mg/kg) were beyond the tolerance of *I. aquatica* in the dry and wet seasons respectively.

**Figure 7:**
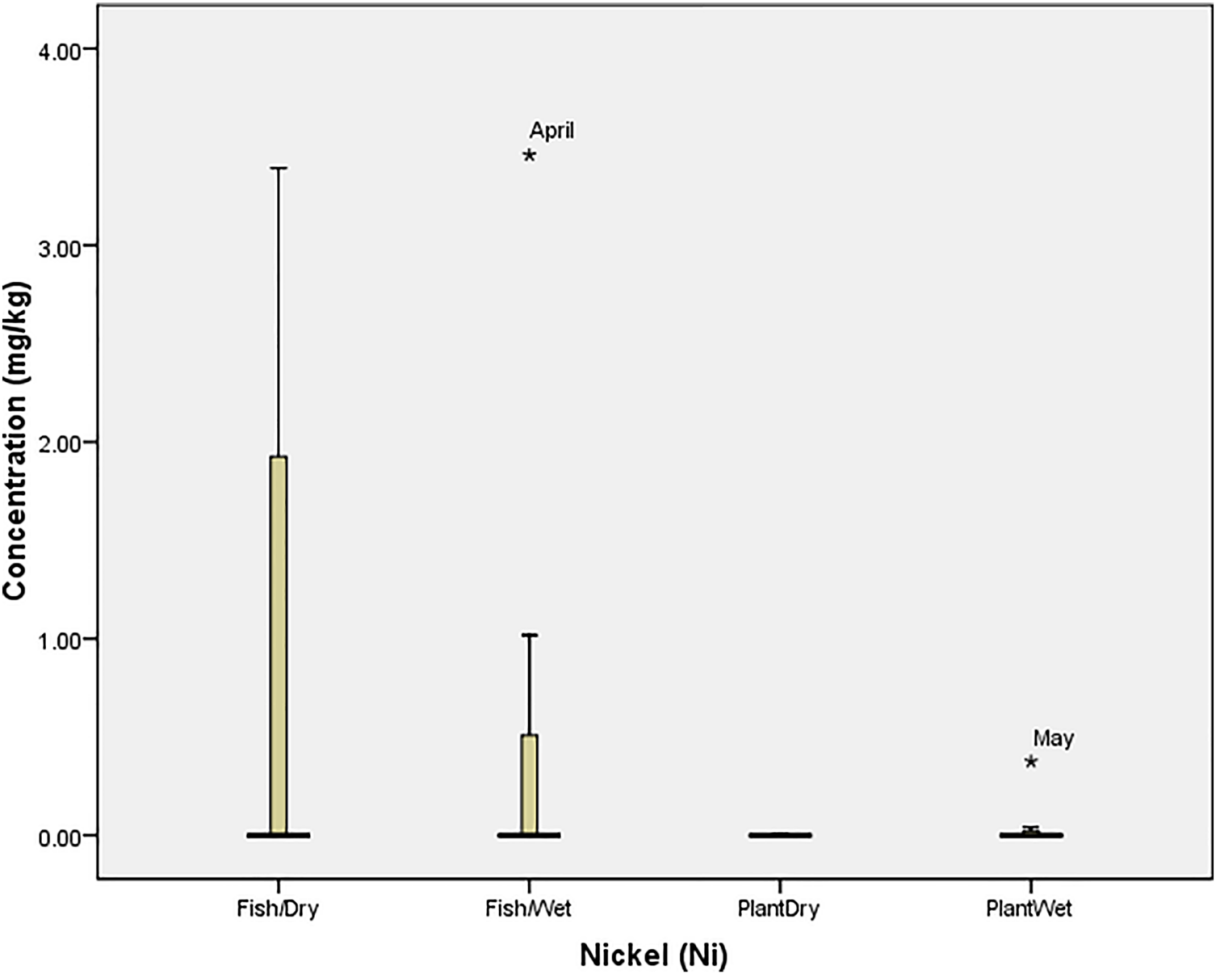
Tolerability of Nickel (Ni) in *O. niloticus* (Fish) and *I. aquatica* (Plant) in Wet and Dry Seasons (Source: Field Survey).

**Figure 8:**
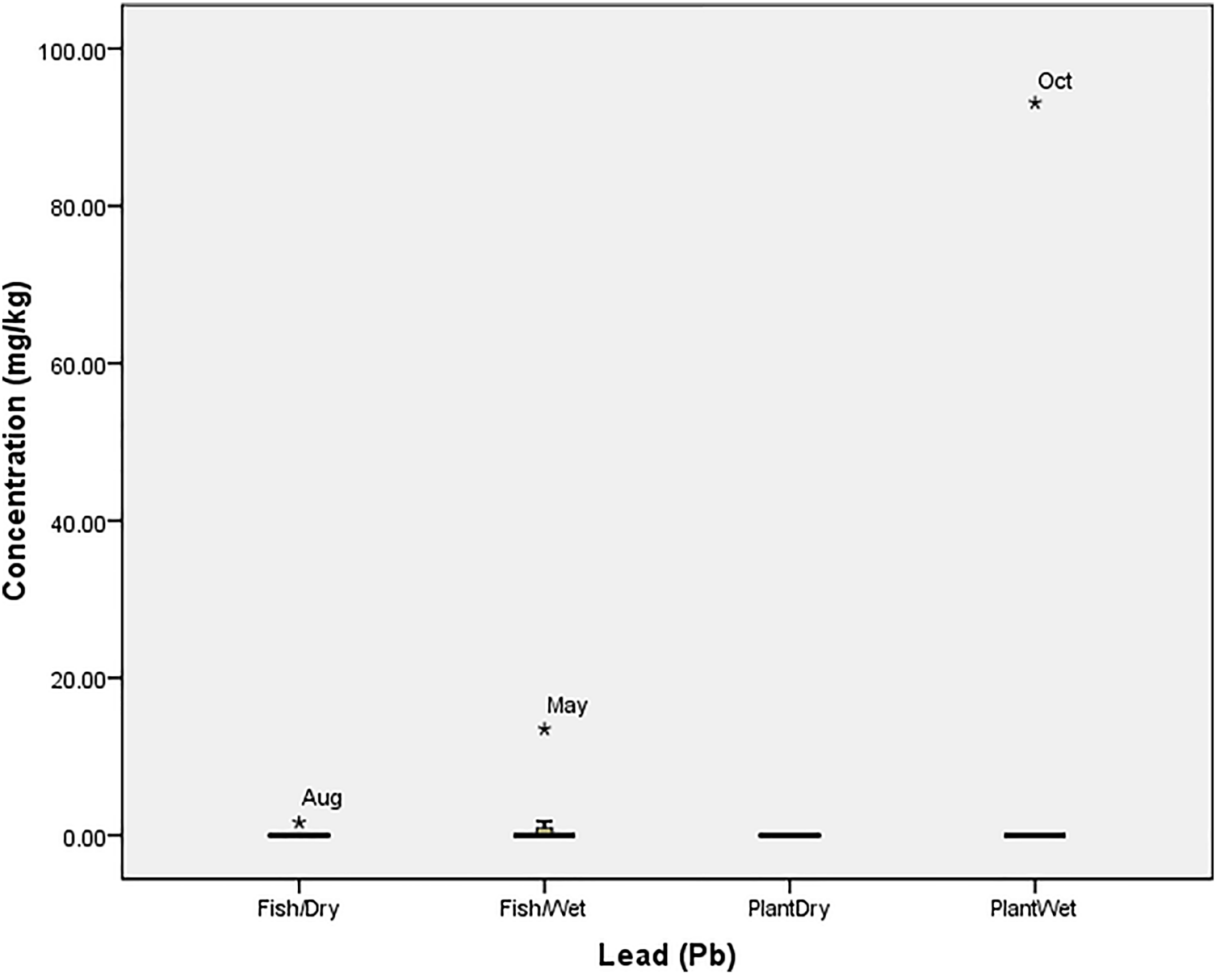
Tolerability of Lead (Pb) in *O. niloticus* (Fish) and *I. aquatica* (Plant) in Wet and Dry Seasons (Source: Field Survey).

**Figure 9:**
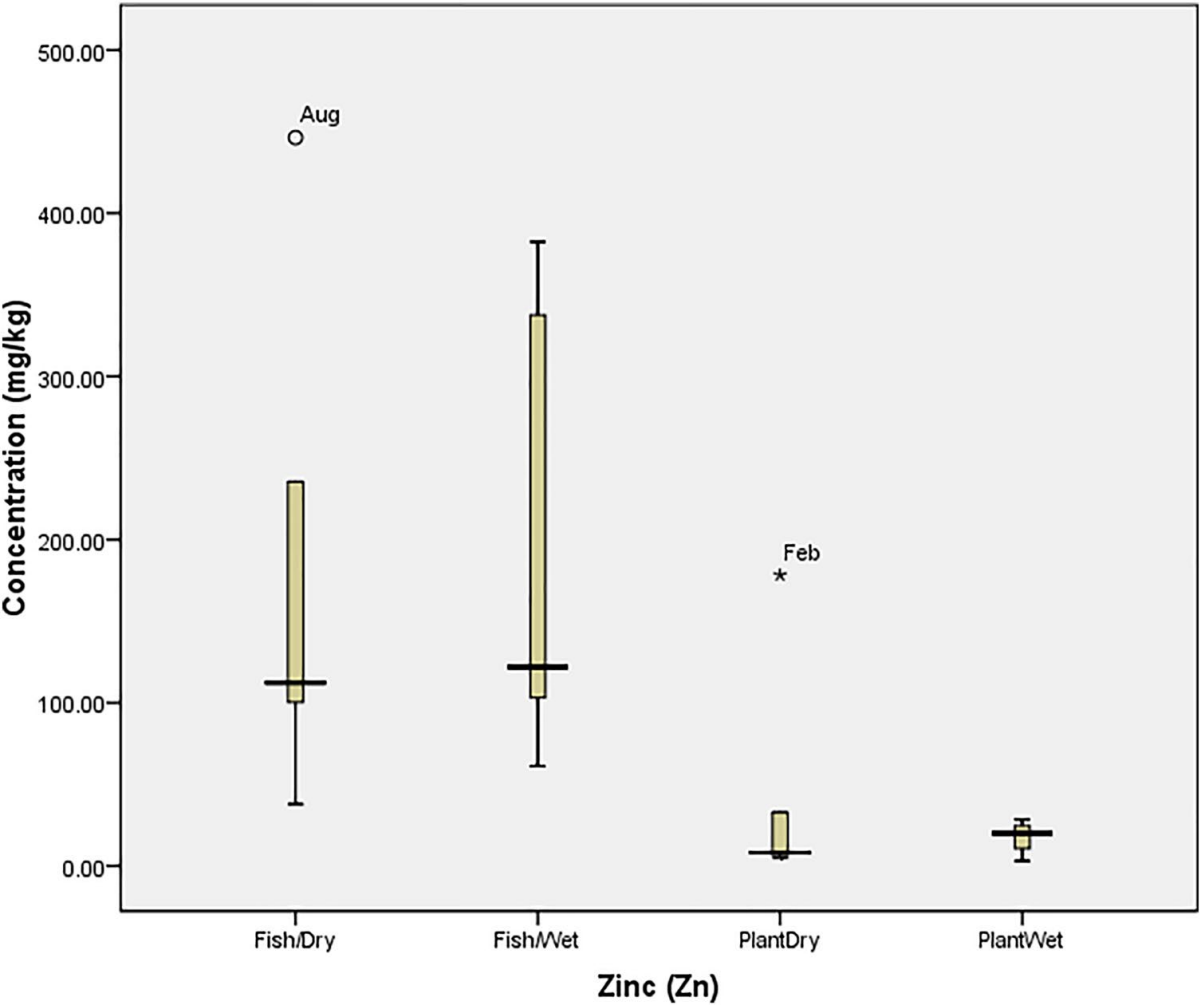
Tolerability of Zinc (Zn) in *O. niloticus* (Fish) and *I. aquatica* (Plant) in Wet and Dry Seasons (Source: Field Survey).

The concentration of nickel (Ni) in April (3.46 mg/kg) and May (0.38 mg/kg) was beyond the tolerance limit of *O. niloticus* and *I. aquatica* respectively (Fig. 7). Also, the concentration of Pb recorded in August (1.61 mg/kg) and May (13.50 mg/kg) was beyond the tolerance limit of *O. niloticus* in the dry and wet seasons respectively while the concentration recorded in October (93.10 mg/kg) was the only toxic concentration in *I. aquatica* (Fig. 8). The high concentrations of Zinc (Zn) recorded in *O. niloticus* were within the tolerance of the fish despite that the concentration recorded in August (446.29 mg/kg) was at the threshold level for the fish. However, the concentration recorded for *I. aquatica* in February (178.27 mg/kg) was beyond the tolerability of the plant (Fig. 9).

## Discussion

The tolerability of heavy metals in the aquatic flora and fauna in Agodi Reservoir was assessed while the obtained metals were evaluated in correspondence with the safety limits of the metal concentrations. Although, some minerals (macro and micro) are required by aquatic plants and animals for efficient growth, development, survival and healthiness of man, exceeding the threshold limit and maximum tolerable intake set by^16^ pose adverse effects to their growth, survival and human health on consumption. Also, *O. niloticus* possess the ability to survive in polluted environmental conditions due to its powerful resistance to disease and slight respiratory demands^17^ as they can accept low oxygen and high ammonia levels, high concentration of metals as observed in this study represents a great threat to the species and fish resources as only Cu did not exceed its tolerability.

*Ipomoea aquatica* has been adduced to be an ecologically abundant leafy vegetable which naturally possesses the ability to bioaccumulate metals from the environment significantly largely from soil and water. The high tolerability of *I. aquatica* to the studied metals was not surprising despite the high concentration of the metals because^18^ had earlier stated that *I. aquatica* readily and naturally absorb heavy metals into their vacuoles while^17^ opined that heavy metals inhibit various enzymes activities and functions in the plant's systems and organs, thus disrupting their metabolic processes especially photosynthesis. Changes in the concentration of metals in aquatic plants have also been adduced to the physicochemical characteristics of the media (soil or water bodies) and the plant's absorption capacity of the plant.

Soils accrue heavy metals in high concentrations and are the major sink for metals that are released into the environment. The use of agrochemicals within the aquatic environment, disposal of both industrial and urban wastes, drainage water from various agricultural practices along the reservoir largely contributes to heavy metal pollution in soil and water, thus contributing to the high concentration in the aquatic fauna and flora^1^.

The ERQ of heavy metals in *I. aquatica* and *O. niloticus* revealed a high ecological risk of Cd, Fe, Zn, Mn and Pb to the aquatic plant and fish as their ERQ values were above the safety limit of one (1). Cd, Pb and Mn, Fe and Zn are highly toxic and harmful metals to the aquatic ecosystem with their accumulation resulting in the deterioration of the ecological balance of the water body.

The high ERQ of the metals observed in both species can result in a reduction in the distribution, composition and dominance of aquatic organisms, changes of aquatic species diversity, biological habitats and entire aquatic ecosystems^19,20^. Also, alterations in the physiological features, biological functions and activities are often experienced. This includes; damages to the respiratory, reproductive, haematological, endocrine, metabolic, digestive and nervous system are often experienced in fish^21,22^. For example, Cd and Pb have been reported to cause the following defects in fish; anaemia and vertebral fractures decreased in the efficiency of the digestive system, persistent behavioural disorders, retardation in growth, erratic swimming, genetic mutation and death^19^.

The distribution of metals across the two bio-indicators was higher in fish than the plant. This corroborates various findings that fish bioaccumulates heavy metals more than aquatic plants ^1,19,23,24^. This is due to the mobility of the fish in the ecosystem, unlike the plant that was rooted to a spot. Also, fishes have the higher tendency and potential to accumulate heavy metals than plants due to their position in the aquatic trophic level as they usually ingest metals from water, aquatic plants and sediments^1,8^.

## Conclusion

This study established that the concentration of heavy metals in fishes and plants inhabiting Agodi reservoir was beyond the recommended limits. The study also revealed that *O. niloticus* bio-concentrated more metals than *I. aquatica.* Consequently, the tolerability of the fish was lower than that of the plant. The ERQ further revealed that both *I. aquatica* and *O. niloticus* constituted ecological risks to the entire aquatic ecosystem and even human consumers due to their abilities to bio-tolerate high concentration of heavy metals. Furthermore, this study could be used as a reference for further research because it provided baseline information on the tolerability of aquatic fauna and flora to heavy metals in a reservoir. This study, therefore, recommends that strict conservation management of the reservoir should be ensured and that the water should be treated (particularly, with focus on removing heavy metals) before being discharged to consumers to protect the aquatic biodiversity and the human population that depends on it.
